# Titanium Dioxide Nanoparticles Induce Maternal Preeclampsia-like Syndrome and Adverse Birth Outcomes via Disrupting Placental Function in SD Rats

**DOI:** 10.3390/toxics12050367

**Published:** 2024-05-16

**Authors:** Haixin Li, Dandan Miao, Haiting Hu, Pingping Xue, Kun Zhou, Zhilei Mao

**Affiliations:** 1 Changzhou Maternity and Child Health Care Hospital, Changzhou Medical Center, Nanjing Medical University, Changzhou 213003, China; sungirllhx@163.com (H.L.); hht1173@126.com (H.H.); 13616118039@163.com (P.X.); 2 Huai’an Center for Disease Control and Prevention, Huai’an 223001, China; amiaodandan@126.com; 3 State Key Laboratory of Reproductive Medicine, Center for Global Health, Nanjing Medical University, Nanjing 211100, China; 4 Key Laboratory of Modern Toxicology of Ministry of Education, School of Public Health, Nanjing Medical University, Nanjing 211100, China; 5 Department of Epidemiology, Center for Global Health, School of Public Health, Nanjing Medical University, Nanjing 211166, China

**Keywords:** titanium dioxide nanoparticles, pregnant model, preeclampsia-like syndrome, autophagy, placenta development, trophoblastic cell function

## Abstract

The escalating utilization of titanium dioxide nanoparticles (TiO_2_ NPs) in everyday products has sparked concerns regarding their potential hazards to pregnant females and their offspring. To address these concerns and shed light on their undetermined adverse effects and mechanisms, we established a pregnant rat model to investigate the impacts of TiO_2_ NPs on both maternal and offspring health and to explore the underlying mechanisms of those impacts. Pregnant rats were orally administered TiO_2_ NPs at a dose of 5 mg/kg body weight per day from GD5 to GD18 during pregnancy. Maternal body weight, organ weight, and birth outcomes were monitored and recorded. Maternal pathological changes were examined by HE staining and TEM observation. Maternal blood pressure was assessed using a non-invasive blood analyzer, and the urinary protein level was determined using spot urine samples. Our findings revealed that TiO_2_ NPs triggered various pathological alterations in maternal liver, kidney, and spleen, and induced maternal preeclampsia-like syndrome, as well as leading to growth restriction in the offspring. Further examination unveiled that TiO_2_ NPs hindered trophoblastic cell invasion into the endometrium via the promotion of autophagy. Consistent hypertension and proteinuria resulted from the destroyed the kidney GBM. In total, an exposure to TiO_2_ NPs during pregnancy might increase the risk of human preeclampsia through increased maternal arterial pressure and urinary albumin levels, as well as causing fetal growth restriction in the offspring.

## 1. Introduction

Titanium dioxide nanoparticles (TiO_2_ NPs) were among the first nanoparticles produced and used worldwide, mainly finding applications in sunscreen, paint, ink, and as a food additive [1,2,3]. TiO_2_ NPs are present in various environmental media, with the primary route of human exposure being through the digestive tract via food [4]. Pregnant women, therefore, cannot avoid exposure to them. Studies have shown that fetuses are more sensitive to toxins than adults, and prenatal exposure can lead to developmental toxicity in offspring [5]. To date, limited human epidemiological evidence regarding the hazards of TiO_2_ NPs to pregnant women and birth outcomes has been reported. Existing studies about the toxicity of TiO_2_ NPs mainly focus on animal models [6,7], making related studies urgently necessary.

Previous research has indicated that TiO_2_ NPs can reach and accumulate in the placenta, leading to a smaller feto-placental unit [8]. They can even penetrate the placental barrier, reach fetal brains, and ultimately affect the offspring’s neurodevelopment [9]. These findings underscore that the placenta is one of the target organs of TiO_2_ NPs. The placenta plays a vital role in embryo development, facilitating substrate exchange, hormone secretion, and immune defense [10]. Trophoblastic cells are a type of cell that plays a crucial role in the development of the placenta during pregnancy, and their migration and invasion ability are crucial for placental development [11]. The impaired function of trophoblastic cells can result in inadequate infiltration of the placenta into the endometrium and a failure to complete spiral artery (SA) remodeling. Placental dysfunction can lead to abortion, fetal growth restriction, intrauterine anoxia, and even fetal death [12,13,14]. Other pregnancy-related diseases, such as preeclampsia and uteroplacental apoplexy, have also been linked to placental dysfunction [15]. However, whether TiO_2_ NPs affect placental development and induce maternal pregnancy diseases and adverse birth outcomes remains unclear and requires further study.

The placental development and trophoblastic cell invasion pattern in rats closely resemble those in humans. Thus, we utilized pregnant rats as an in vivo model to investigate the potential effects of TiO_2_ NPs on placental development and to uncover the potential underlying mechanisms. Rats have a gestation period of 21 days, with embryos being implanted around 4–5 days after fertilization. Therefore, we selected the 5th day of pregnancy as the starting point for exposure. Throughout this study, we closely monitored maternal changes and recorded pregnancy outcomes after TiO_2_ NPs exposures, with a particular emphasis on observing placental development to elucidate the potential mechanisms at play.

## 2. Materials and Methods

### 2.1. Animals

The animal study was conducted in compliance with the ethical guidelines set forth by the Nanjing Medical University Ethics Committee (Approved No: IACUC-24040115) and followed the principles outlined in the ARRIVE Guidelines for reporting in vivo experiments. Titanium dioxide nanoparticles (TiO_2_ NPs, CAS number: 13463-67-7) were purchased from Sigma-Aldrich(Sigma Chemical Co., St. Louis, MO, USA). Adult male and female SD rats (8 weeks, 280–300 g) were obtained from Beijing Vital River Laboratory Animal Technology Co., Ltd. All rats were housed separately, by gender, and acclimatized in a controlled environment maintained at a temperature of 22 ± 2 °C and humidity of 40–60%, with a 12 h light/dark cycle, for one week of rest prior to the commencement of the experiments. Female rats were paired with males in a 1:1 ratio following random grouping, with 10 rats in each group. Male rats were separated from females after confirming the presence of a vaginal plug every morning, and this was recorded as gestational day 0.5 (GD 0.5). 

### 2.2. Cell Culture

Trophoblastic cells (HTR8-Svneo) were purchased from American Type Culture Collection (ATCC^®^ CRL-3271™) and cultured in Roswell Park Memorial Institute 1640 (RPMI-1640) medium supplemented with 10% fetal bovine serum (FBS), 100 U/mL penicillin, and 100 μg/mL streptomycin. The culture dishes were incubated in a 37 °C, 5% CO_2_ atmosphere, and the medium was replaced every day.

### 2.3. TiO_2_ NPs Preparation and Exposure Design

The TiO_2_ NPs were dispersed in a 5% methylcellulose solution at a concentration of 5 mg/mL. Their characteristics were assessed following sonication at 100 W for 30 min. A morphological analysis was conducted using transmission electron microscopy (TEM), while their hydrodynamic diameter was determined using dynamic light scattering (DLS). To mimic human exposure routes and doses, pregnant rats were orally administered TiO_2_ NPs at a dose of 5 mg/kg body weight per day from GD5 to GD18 during pregnancy. Control rats received treatment with a 0.5% methylcellulose solution [16]. The dose of 5 mg/kg/day was selected based on the average daily human consumption of TiO_2_ [17]. Following delivery, except for those euthanized for further analysis, the remaining female rats ceased their TiO_2_ NP exposure. Throughout the study, all pregnant rats were monitored for weight changes before supplying food. 

### 2.4. Tissue Collection and Preparation

The pregnant rats were euthanized using 10% chloral hydrate on gestational day 18 (GD 18). Subsequently, maternal organs including the liver, spleen, kidney, and placenta were carefully dissected, counted, and weighed to calculate the organ coefficients (weight of the organ/total body weight) and then preserved in 4% polyformaldehyde for subsequent analysis. Pregnancy outcomes including fetal numbers and fetal growth conditions were documented simultaneously. To prevent the separation of the placenta from the uterus, the placenta–uterus units were carefully kept intact, ensuring the preservation of the placental invasion ability for subsequent analyses.

### 2.5. Histopathological Analysis and Immunohistochemical Analysis

The tissues were fixed and dehydrated before being embedded in paraffin. Subsequently, tissue blocks were sectioned into slices. These slices underwent dewaxing with xylene, followed by rehydration with graded alcohol. Hematoxylin and eosin (HE) staining and periodic acid-Schiff (PAS) staining were performed using a commercial kit and antibody (Beyotime, C0105, Shanghai, China; Abcam, ab150680, Cambridge, UK), following the manufacturer’s instructions. PAS staining was employed to highlight the elastic fibers, collagen, and other components in the kidney. The immunohistochemical analysis was conducted using an anti-LC3B antibody (Abcam, ab48394) in conjunction with an HRP-conjugated secondary antibody. All sections were examined under a light microscope, and images were semi-quantified using ImageJ Software 1.8.0 (National Institute of Health, USA).

### 2.6. Placenta Invasion Ability Assessment

The placental invasion ability assessment was conducted following the methodology outlined in a previous study [18]. The percentage of interstitial trophoblast invasion into the mesometrial triangle (MT) was utilized to quantify the invasion ability, with infiltrated trophoblast cells identified using a cytokeratin-7 (CK-7) antibody (Abcam, ab181598). Evidence of spiral artery (SA) remodeling was also identified through the presence of CK-7-positive cells arranged on a fibrinoid layer, alongside the absence of α-actin-positive smooth muscle cells. Additionally, the cross-sectional area, as reported by Cotechini et al., was included as an informative indicator [19]. Both the ratio of the cytokeratin-7-positive trophoblast cell area to the MT area and the cross-sectional areas were measured using image J analysis software.

### 2.7. Immunofluorescence Analysis

A trophoblastic cell (HTR8-Svneo) model was employed for in vitro mechanism verification. Cellular autophagy levels were assessed using an anti-LC3B antibody in conjunction with a confocal microscope. Cells were seeded onto specialized dishes and incubated with 10 μg/mL TiO_2_ NPs. After exposure for 24 h, cells were fixed and treated with the primary antibody overnight, followed by a CY3-labeled secondary antibody. Images were captured using a confocal microscope system.

### 2.8. Cell Invasion and Migration Ability Analysis

Cell invasion and migration ability were evaluated using a transwell assay. In brief, HTR cells were exposed to 10 μg/mL TiO_2_ NPs and suspended in serum-free medium. These cells were then seeded onto matrigel-coated upper chambers, while a serum-containing medium was added to the lower chambers. After a 24 h incubation period, the cells were fixed and stained with crystal violet, and the number of penetrated cells was counted using a light microscope.

### 2.9. Maternal Blood Pressure Monitoring

Maternal mean arterial pressure (MAP) was assessed using a non-invasive blood pressure analyzer on GD0 (before mating), GD18, and the third day after delivery (AD3) in both experimental groups. The research was conducted in a controlled environment to minimize noise, with room temperature maintained between 25 and 26 °C. Female rats were gently restrained, and the pressure detector was securely positioned on their tails. Once the animals had calmed for approximately 3 min, the measurements were initiated. Each rat underwent 6 consecutive series of measurements, and any aberrant data points were excluded from the analysis.

### 2.10. Determination of Proteinuria

Spot urine samples from female rats in both experimental groups were collected at the corresponding time points to assess the occurrence of proteinuria, as previously described [20]. Urinary albumin and urine creatinine concentrations were quantified using commercial assay kits (TRFIA) (Lumigenx, Suzhou, China) following the manufacturer’s instructions. The albumin to creatinine ratio (ACR) was utilized as an indicator of proteinuria.

### 2.11. Statistical Analysis

Statistical analyses were performed using SPSS software(IBM, Armonk, NY, USA). The normality and homogeneity of variance for all data were assessed using the Kolmogorov–Smirnov test. Quantitative data were presented as mean ± SD. The comparison of differences between two groups or among multiple groups was conducted using the *t*-test and a one-way ANOVA, respectively. The difference between the two ratios was assessed using the Chi-square test. A *p*-value of less than 0.05 was considered statistically significant. 

## 3. Results

### 3.1. Main Characteristics of TiO_2_ NPs

**Figure 1:**
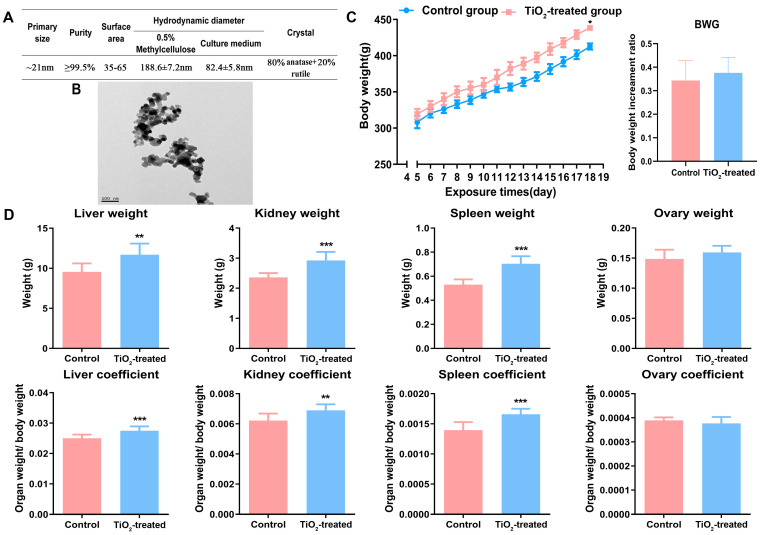
(**A**,**B**) The characteristics of TiO_2_ NPs determined by a transmission electronic microscope (TEM), dynamic light scattering (DLS), and by the manufacturer’s report. (**C**) Maternal body weights were determined before the female rats were fed every morning. Body weight gain (BWG) = (mf − mi)/mi. “mf” represents the final body weight and “mi” represents the initial body weight. (**D**) The main organs (liver, kidney, spleen, and ovary) were weighed and the organ coefficients were calculated after the pregnant rats were executed on GD18. * *p* < 0.05, ** *p* < 0.01, *** *p* < 0.001. There were 7 rats in the control group and 8 rats in the exposure group.

The main characteristics of TiO_2_ NPs in 0.5% methylcellulose and in cell culture medium were determined and are presented in Figure 1A. The transmission electron microscopy (TEM) results (Figure 1B) revealed that the morphology of TiO_2_ NPs was nearly spherical, with a primary size of approximately 21 nm. The purity of the TiO_2_ NPs was reported to be ≥99.5% in terms of trace metals, with a BET surface area ranging from 35 to 65 m^2^/g, and their crystal form was determined to be 80% anatase and 20% rutile according to the manufacturer’s reports. Additionally, the results of dynamic light scattering (DLS) indicated that the average hydrodynamic diameter of the TiO_2_ NPs was approximately 190 nm in 0.5% methylcellulose and about 80 nm in complete cell culture medium containing serum, which is consistent with our previous study.

### 3.2. Effects of TiO_2_ NPs on Maternal Conditions

The results revealed that the conception rate, with both groups showing 7 or 8 out of 9 successful conceptions, exhibited no difference after the observation of vaginal plugs. Maternal body weight and organ weights were recorded before and after the TiO_2_ NPs exposure. The monitoring of body weight indicated that the maternal body weight did not differ at the beginning of the study but increased throughout the entire pregnancy period in both groups, with a significant difference observed at the end stage of pregnancy, while the body weight gain (BWG) showed no significant change (Figure 1C). Additionally, compared to control rats, the weight of the maternal liver, kidney, spleen, and their corresponding organ coefficients, all increased on GD18 following exposure. Conversely, the weight of the ovary and the ovarian coefficient showed no significant change between the two groups on GD18 (Figure 1D).

### 3.3. Pathological Changes of Maternal Organs after TiO_2_ NPs Exposure

**Figure 2:**
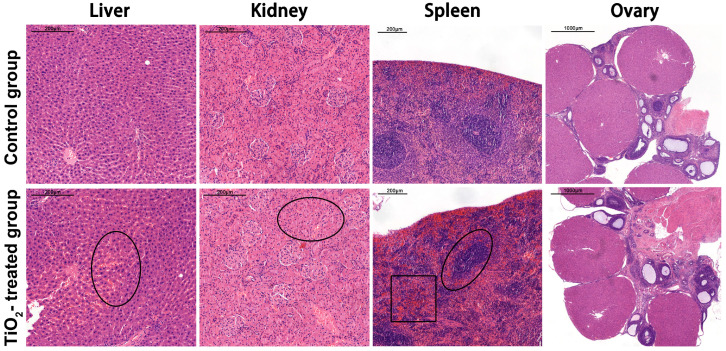
The pathological changes in the maternal liver, kidney, spleen, and ovary after exposure were examined by light microscope after hematoxylin and eosin (HE) staining. Scale bar = 200 μm in the liver, kidney, and spleen. Scale bar = 1000 μm in the ovary. Pathological changes in the liver, kidney, and spleen were indicated with black circles, and hyperemia region was indicated with black square. There were 7 rats in the control group and 8 rats in the exposure group.

The maternal liver, kidney, spleen and ovary were examined by hematoxylin–eosin (HE) staining. Compared with the control group, hyperemia occurred in the liver after exposure, resulting in an edema and the degeneration of liver cells in the hyperemic areas, with degenerative particles appearing in the cytoplasm. This occurred partly, rather than diffusing throughout the entire organ. After the TiO_2_ NPs exposure, we observed that the volume of glomeruli increased and the renal tubular cells exhibited edema. The maternal splenic corpuscles either disappeared or were demolished, with splenic sinusoids exhibiting hyperemia. The area of white pulp decreased, indicating atrophy of the white pulp. Additionally, the splenic marginal zone widened. In comparison to the control group, the maternal ovary showed no pathological changes after exposure (Figure 2).

### 3.4. Effects of TiO_2_ NPs on Fetal Birth Outcomes

**Figure 3:**
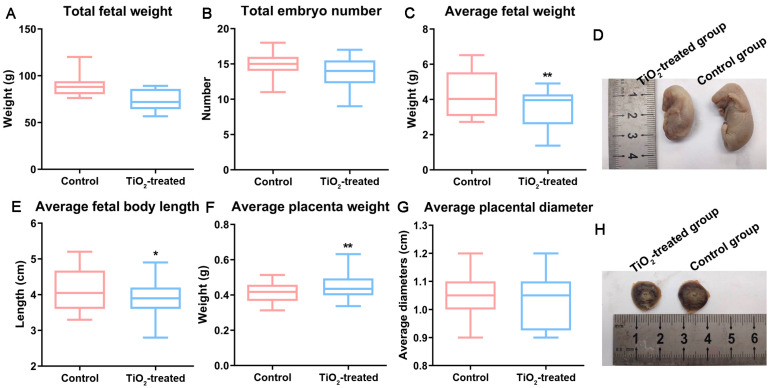
The main birth outcomes were recorded on GD18 after the mothers were executed. The total fetal weight (**A**), total embryo number (**B**), average fetal weight (**C**), average fetal body length (**E**), average placental weight (**F**), and placental diameter (**G**) were obtained from the control and TiO_2_-treated group. (**D**,**H**) show images of the fetuses and placenta. * *p* < 0.05, ** *p* < 0.01. There were 105 fetal rats in the control group and 95 fetal rats in the exposure group.

Compared with the control group, the early embryo resorption rate significantly increased after an exposure to TiO_2_ NPs, with a ratio of 0.019 (2/105) in the control group and 0.0737 (7/95) in the exposure group. Moreover, the number of pregnant rats experiencing embryo loss significantly increased (2/7 in the control group and 5/8 in the exposure group). Even a monocyesis was observed after exposure, which barely happens during normal pregnancy (Figure S1). Although there was a decreasing trend, the total number and total weight of the fetuses (including the fetus, placenta, and uterus) did not show a significant difference after their exposure to TiO_2_ NPs (Figure 3A,B). However, the average body weight (Figure 3C) and body length (Figure 3D,E) of the fetal rats decreased significantly (*p* < 0.05). The average placental diameters showed no difference (Figure 3G,H), while their corresponding weights exhibited a slight but significant increase (0.05 g) in the exposure group (Figure 3F). 

### 3.5. TiO_2_ NPs Increased Maternal Mean Arterial Pressure (MAP)

**Figure 4:**
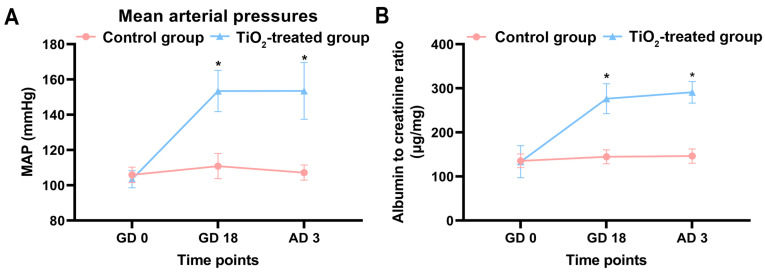
(**A**) The average maternal arterial pressures were measured using a non-invasive blood pressure analyzer before pregnancy (GD0), on the 18th day of gestation (GD18), and on the third day after delivery (AD3). (**B**) The maternal urinary protein levels were determined using spot urine samples, and the urinary albumin to creatinine ratio (ACR) was utilized to normalize the proteinuria. The data were indicated as mean ± SD. * *p* < 0.05. There were 7 rats in the control group and 8 rats in the exposure group.

Maternal mean arterial pressures (MAPs) were measured on GD0 (before mating), GD18, and on the third day after delivery (AD3). The blood pressure monitoring results indicated no significant difference between the two groups on GD0. However, the MAP significantly increased in the exposure group on GD18 (Figure 4A). Furthermore, even after delivery, their maternal MAPs remained significantly higher than those of the control rats, indicating irreversible damage to the mothers.

### 3.6. TiO_2_ NPs Induced Maternal Proteinuria

Maternal proteinuria was assessed on GD0, GD18, and AD3. The findings revealed that, after adjusting for the effects of creatinine, the albumin-to-creatinine ratio (ACR) significantly increased in the TiO_2_ NPs exposure group on GD18, indicating the occurrence of proteinuria (Figure 4B). Moreover, the proteinuria persisted on the third day after delivery, showing no signs of diminishing.

### 3.7. Effects of TiO_2_ NPs on Placental Infiltration into Uterus

**Figure 5:**
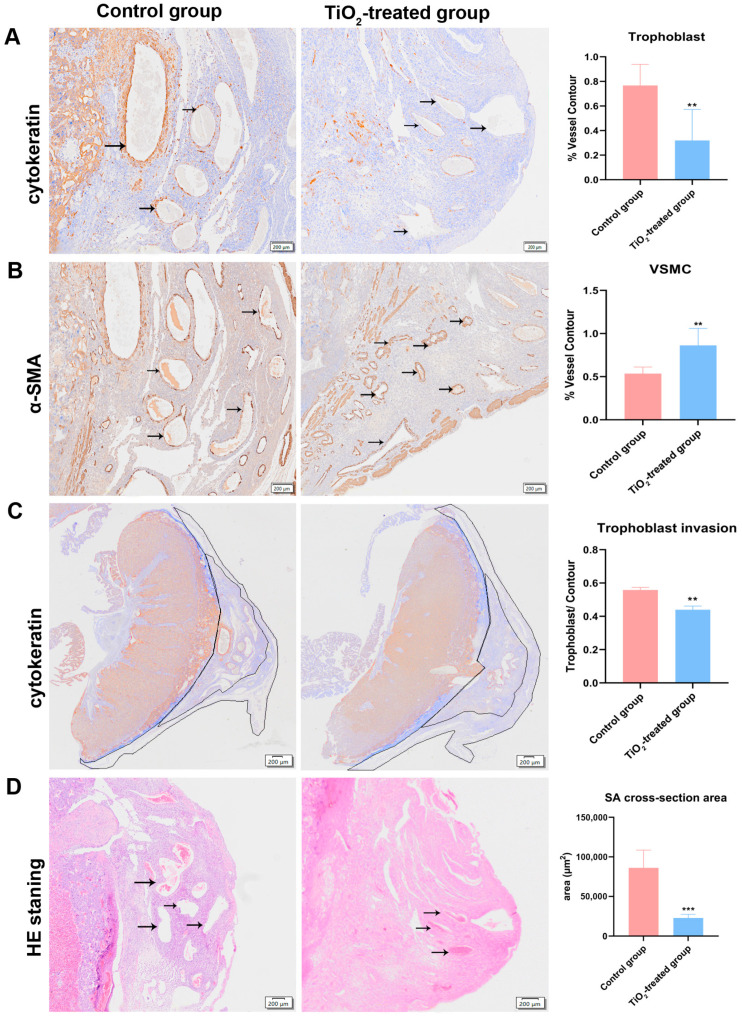
(**A**) A placental invasion ability assessment was conducted using the percentage of interstitial trophoblast invasion into the mesometrial triangle (MT). The infiltrated trophoblast cells were identified using a cytokeratin-7 (CK-7) antibody. (**B**) Evidence of spiral artery (SA) remodeling was also identified through the α-actin-positive smooth muscle cells. (**C**) The ratio of the cytokeratin-7-positive trophoblast cell area to the MT area. (**D**) The cross-sectional areas and SA numbers were measured by HE staining. A quantitative analysis was carried out with the Olympus OlyVIA software. The data were indicated as mean ± SD. ** *p* < 0.01, *** *p* < 0.001. The obvious pathological changes were indicated with black arrows, and the trophoblast invasion areas were outlined. There were 7 rats in the control group and 8 rats in the exposure group.

Placental infiltration and spiral artery (SA) remodeling were assessed using image analysis software after revealing interstitial trophoblastic cells and smooth muscle cells. The immunohistochemistry results indicated a decreased ratio of cytokeratin-positive and an increased ratio of actin-positive cells in SA (Figure 5A,B). The area percentage of interstitial trophoblast invasion into the maternal tissue (MT) significantly decreased after the maternal exposure to TiO_2_ NPs (Figure 5C). Additionally, a reduction in both the number and cross-section areas of the spiral arteries (SAs) in the placental triangle was observed in the TiO_2_ NPs group compared to the control group (Figure 5D).

### 3.8. Effects of TiO_2_ NPs on Maternal Glomerular Basement Membrane (GBM)

**Figure 6:**
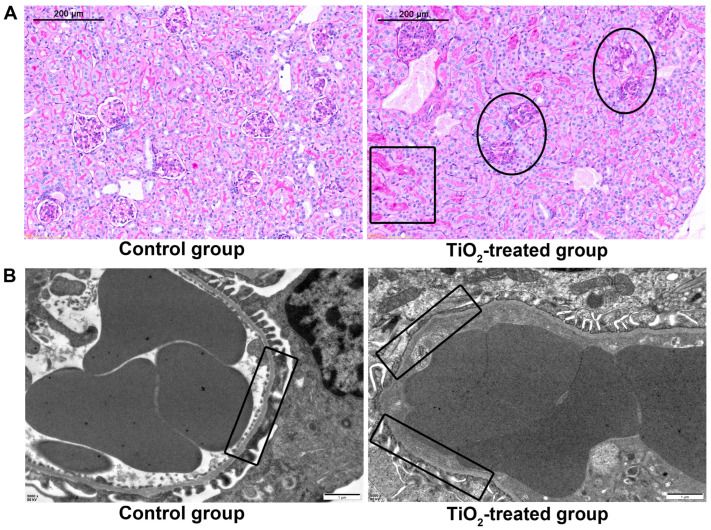
(**A**) The glomerular basement membrane (GBM) and deposit of fibrin were revealed by periodic acid-Schiff (PAS) staining. (**B**) The ultra microstructure of the GBM was revealed by TEM after a series of sample preparations, bar = 1 μm. The glomerular lesions were indicated with black circles, and the fibrin deposition region was indicated with a black square. The normal and impaired GBM were highlighted with black squares in TEM images. There were 7 rats in the control group and 8 rats in the exposure group.

This glomerulus was stained with PAS to reveal the basement membranes. As shown in Figure 6A, after the TiO_2_ NPs exposure, the capillary loops of the glomerulus were thin and well defined, while the loops were blurred and the normal structures had disappeared, indicating that the GBM was destroyed. A fibrinous deposition was easily observed in the glomerulus. Figure 6B showed the ultra-microstructure observed by TEM; as shown, the control tissue showed a clear and consecutive GBM, while the GBM in the exposure group was fuzzy and became thin, fractures even occurred in certain areas.

### 3.9. Effects of TiO_2_ NPs on the Migration and Invasion Ability of Human Trophoblastic Cells

**Figure 7:**
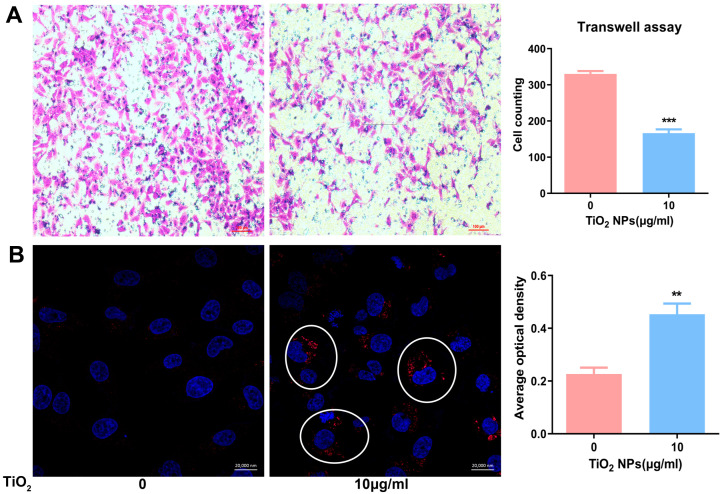
(**A**) The cellular migration and invasion ability of trophoblastic cell lines (HTR) were determined by a transwell assay; cell counting was obtained from five independent fields of the light microscope, and the data were presented as mean ± SD, scale bar = 100 μm. (**B**) The autophagy levels of HTR cells exposed to TiO_2_ NPs were examined by immunofluorescence; the nuclei were stained blued with DAPI and the autophagosomes were stained red with CY3. Their fluorescence density was measured with the Zeiss software (https://www.zeiss.com/microscopy/en/products/software/zeiss-zen.html accessed on 9 May 2024) within the laser confocal microscope software package. Scale bar = 20,000 nm. *** *p* < 0.001, ** *p* < 0.01. The autophagsomes are indicated by the white circles in the image.

The migration and invasion abilities of human trophoblastic cells were assessed using a transwell assay. The results (Figure 7A) revealed that, after an exposure to 10 μg/mL TiO_2_ NPs, the number of penetrated cells significantly decreased when observed under a light microscope.

### 3.10. Effects of TiO_2_ NPs on the Autophagy of Human Trophoblastic Cells

The autophagy levels of HTR cells were assessed through immunofluorescence combined with a confocal microscope examination following their exposure to 10 μg/mL TiO_2_ NPs. Figure 7B showed that, after the exposure of TiO_2_ NPs, there was an observed increase in the autophagy level of HTR cells. Specifically, in the control cells, there were few LC3-positive dots, whereas, in the treated group, numerous autophagosomes formed and accumulated in the cytoplasm.

## 4. Discussion

Due to the widespread use of TiO_2_ NPs in everyday products and the indications of their toxicity to humans from numerous animal studies [21,22], concerns regarding their safety for human beings, particularly pregnant women, have been raised. However, to date, there is limited epidemiological evidence proving the risks of TiO_2_ NPs to pregnant women and their fetuses. Therefore, establishing pregnant animal models is essential to explore the potential effects following from TiO_2_ NPs exposures. In a previous study, Yamashita et al. established a pregnant mice model to investigate the adverse effects of TiO_2_ NPs on maternal and fetal health. They found that the placenta played a significant role as a target organ in mediating toxicity during pregnancy [23]. However, it has been suggested that the rat placenta may be more suitable for studying human placental function, considering the similarities in its trophoblastic cell invasion and spiral arterial remodeling [24]. In this study, we opted for a pregnant rat model as it represents a suitable model and we primarily focused on placental development after TiO_2_ NPs exposures.

To our knowledge, pregnant females are sensitive to their surroundings and to different manipulations, which can sometimes lead to abortion during the early stages of pregnancy [25]. However, our results indicated that the conception rate did not differ between the two groups, suggesting that the effects of manual manipulation could be disregarded. We observed that the maternal body weight across the two groups significantly increased from the 12th day. We attribute this finding to the complex changes involving organ weights and fetal weights, as well as the enhanced maternal blood glucose level reported in our previous study [26]. Specifically, major maternal organs such as the liver, kidney, and spleen exhibited hyperemia, with both organ weight and organ coefficient showing significant increases. Meanwhile, the total fetal weight decreased, a phenomenon also reported in Hong’s study [27].

Previous studies evaluating TiO_2_ NPs’ toxicity have shown similar pathological changes in the liver, with researchers attributing these changes to inflammatory responses or disturbances in the antioxidant system [28]. The kidney, which is the main organ for TiO_2_ NPs’ excretion [29], exhibited glomerular swelling and an accumulation of red blood cells after exposure, consistent with previous findings [30]. This alteration may result from inflammation, as similar results have been reported in other studies [31]. The spleen, an important immune organ, contains numerous macrophages at its edges, which play a crucial role in responding to foreign substances [32]. In our study, we observed a widening of the spleen edge after exposure, indicating a vigorous immune response, consistent with previous findings that TiO_2_ NPs prime a specific activation state of macrophages [33]. Additionally, a previous study demonstrated that TiO_2_ NPs exert immune toxicity by inducing apoptosis and Toll-like receptor signaling [34], a finding supported by the observed atrophy of the white pulp in our study. 

Researchers have found that TiO_2_ NPs can induce premature ovarian failure, follicle disorders, and ovarian dysfunction in female mice [35,36,37], indicating reproductive toxicity in this species. However, no obvious pathological changes were observed in the present study. This might be explained by the fact that, during pregnancy, follicle development halts and its blood supply decreases compared to that in non-pregnant females, potentially reducing the impact on the ovary, similar to the kidney responses.

Following the exposure to TiO_2_ NPs, there was a significant increase in embryo loss, consistent with a previous finding [38]. Our results, along with those of previous reports, suggest that TiO_2_ NPs may increase the abortion rate in humans after maternal exposure. Decreases in average body weight and length suggest inhibited fetal development, raising concerns about the potential associations between TiO_2_ NPs exposure and low fetal birth weight or fetal growth restriction (FGR) in humans. As far as our knowledge extends, the placenta plays a crucial role in embryo development, and the failure of proper placental invasion can lead to abnormal fetal development. Therefore, placental infiltration was further evaluated in our study. 

The migration and invasion of trophoblast cells are known to be associated with the maternal vascular network [39], and the invasion of trophoblast cells into the spiral arteries is a critical process for vascular remodeling during pregnancy. Therefore, we evaluated indicators closely related to placental development, including the percentage of the area invaded by trophoblast cells in the mesometrial triangles, the presence of trophoblast cells resting on a fibrinoid layer [18], and the cross-sectional area of the spiral arteries [19]. Our results suggested an insufficient invasion of the placenta into the endometrium and a failure to complete spiral artery remodeling. This restricted fetal blood supply and affected fetal development, resulting in decreased fetal weight and length in the exposure group, resembling the progression of preeclampsia.

Inflammatory responses, cell apoptosis, and reactive oxygen species (ROS) were observed in a placenta model after a maternal exposure to TiO_2_ NPs [40,41]. The authors suggested these as possible mechanisms for placental vascular dysfunction. Recent studies have shown that autophagy is an important mechanism contributing to placental dysplasia [42]. Additionally, TiO_2_ NPs have been reported to induce autophagy in various cell lines [43], potentially inhibiting the normal development of the rat placenta by inducing autophagy in trophoblastic cells. Therefore, we investigated whether autophagy plays a crucial role in TiO_2_ NPs-induced placental dysfunction. Our results showed a significant increase in autophagy levels in the labyrinthine placenta after exposure. The evidence indicates that TiO_2_ NPs can accumulate in the placenta and reach relatively high levels even after exposure during pregnancy [27]. To verify whether TiO_2_ NPs could induce autophagy at relatively low doses, we administered 10 μg/mL of TiO_2_ NPs to HTR, a human-derived cell line, for further study. The results confirmed that TiO_2_ NPs could induce cell autophagy and inhibit the migration and invasion ability of human trophoblastic cells, validating the results observed in animal studies and raising concerns about the risks to pregnant women. 

Considering that TiO_2_ NPs induced significant pathological and functional changes in both the kidney and placenta, resembling those seen in preeclampsia, our study observed increased blood pressure and the appearance of proteinuria after exposure, on GD18. Previous animal studies suggested that TiO_2_ NPs may deposit on the glomerular basement membrane, inducing kidney inflammation, although the total urine protein levels did not significantly change [44]. In our study, proteinuria was detected, potentially indicating an increased kidney burden during pregnancy and protein leakage. It has been reported that nanoparticles can induce kidney injury and elevate urinary retinol-binding proteins, supporting our findings [45]. The kidney plays a crucial role in blood pressure regulation [46], and TiO_2_ has been shown to activate ROS [47] or induce fibrosis via the Wnt pathway [48], thus contributing to increased blood pressure through renal impairment via reported or undetermined pathways after a TiO_2_ NPs exposure. An important characteristic of preeclampsia is the resolution of hypertension and proteinuria after delivery. However, in our study, hypertension and proteinuria did not revert to pre-pregnancy levels. It is important to note that the disease induced by TiO_2_ NPs is not traditional preeclampsia, and treatments for preeclampsia may not be effective and could potentially exacerbate these related clinical symptoms during pregnancy. In our previous work, we identified targets for reversing autophagy to mitigate the adverse effects of TiO_2_ NPs during human placental development [49]. However, considering that microRNAs are species-specific and the candidate microRNAs identified in human trophoblastic cells may not be suitable for use in rats, we were unable to observe birth outcomes and placental development after reversing placental autophagy. Nonetheless, targeting autophagy reversal may be a useful approach to alleviating TiO_2_ NPs-related symptoms in pregnant women.

## 5. Conclusions

In this study, we demonstrated that pregnant rats exposed to TiO_2_ NPs via their digestive tract from GD5 to GD18 exhibited significant alterations in maternal physiology, organ pathology, fetal growth restriction, and a maternal preeclampsia-like syndrome. The adverse effects on the fetus and mother may be associated with placental dysplasia, which may be related to deficiencies in autophagy-related cell migration and invasion. Overall, TiO_2_ NPs induced symptoms resembling those of preeclampsia in pregnant rats.

## Data Availability

The data presented in this study are available on request from the corresponding author.

## Supplementary Materials

The following supporting information can be downloaded at https://www.mdpi.com/article/10.3390/toxics12050367/s1, Figure S1: The image of the maternal uterus that carried single pregnancy.
