# Supplementary figures and images for: Titanium Dioxide Nanoparticles Induce Maternal Preeclampsia-like Syndrome and Adverse Birth Outcomes via Disrupting Placental Function in SD Rats
Source: Toxics. 2024 May 16;12(5):367. doi: 10.3390/toxics12050367 (PMC11125676; doi:10.3390/toxics12050367)

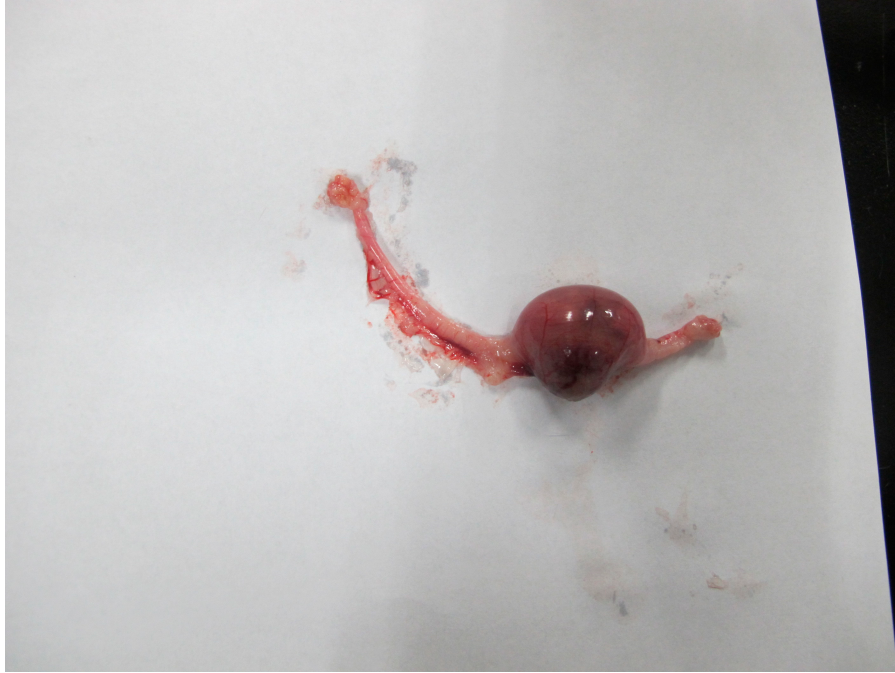

Figure S1: The image of the maternal uterus that carried single pregnancy.

Supplement: Supplementary file 1 [file toxics-12-00367-s001.zip › toxics-2999096-supplementary.pdf]
